# Supplementary material for: A multi-center cross-sectional investigation of BRAF V600E mutation in Ameloblastoma
Source: PeerJ. 2025 Apr 3;13:e19137. doi: 10.7717/peerj.19137 (PMC11972564; doi:10.7717/peerj.19137)
Supplement: Supplemental Information 3 [file peerj-13-19137-s003.docx]

STROBE Statement—Checklist of items that should be included in reports of ***cross-sectional studies***

|  | Item No | Recommendation | Checklist |
| --- | --- | --- | --- |
| **Title and abstract** | 1 | (*a*) Indicate the study’s design with a commonly used term in the title or the abstract | P1, line 1-3  P5, line 33 |
|  |  | (*b*) Provide in the abstract an informative and balanced summary of what was done and what was found | P5, line 36-52 |
| Introduction | | |  |
| Background/rationale | 2 | Explain the scientific background and rationale for the investigation being reported | P6-9, line 60-94 |
| Objectives | 3 | State specific objectives, including any prespecified hypotheses | P9, line 94-98 |
| Methods | | |  |
| Study design | 4 | Present key elements of study design early in the paper | P10, line 102-104 |
| Setting | 5 | Describe the setting, locations, and relevant dates, including periods of recruitment, exposure, follow-up, and data collection | P10, line 116-121 |
| Participants | 6 | (*a*) Give the eligibility criteria, and the sources and methods of selection of participants | P10, line 121-126 |
| Variables | 7 | Clearly define all outcomes, exposures, predictors, potential confounders, and effect modifiers. Give diagnostic criteria, if applicable | P11, 12, line 138-144 |
| Data sources/ measurement | 8* | For each variable of interest, give sources of data and details of methods of assessment (measurement). Describe comparability of assessment methods if there is more than one group | P10, line 116-118 |
| Bias | 9 | Describe any efforts to address potential sources of bias | P11, line 138-141 |
| Study size | 10 | Explain how the study size was arrived at | P10, line 110-118 |
| Quantitative variables | 11 | Explain how quantitative variables were handled in the analyses. If applicable, describe which groupings were chosen and why | Not relevant |
| Statistical methods | 12 | (*a*) Describe all statistical methods, including those used to control for confounding | P16, line 174-179 |
|  |  | (*b*) Describe any methods used to examine subgroups and interactions | P16, line 175-177 |
|  |  | (*c*) Explain how missing data were addressed | Not relevant |
|  |  | (*d*) If applicable, describe analytical methods taking account of sampling strategy | Not relevant |
|  |  | (*e*) Describe any sensitivity analyses | Not relevant |
| Results | | |  |
| Participants | 13* | (a) Report numbers of individuals at each stage of study—eg numbers potentially eligible, examined for eligibility, confirmed eligible, included in the study, completing follow-up, and analysed | P17, line 183  P20, line 209, P22, line 226 |
|  |  | (b) Give reasons for non-participation at each stage | Not relevant |
|  |  | (c) Consider use of a flow diagram | Not relevant |
| Descriptive data | 14* | (a) Give characteristics of study participants (eg demographic, clinical, social) and information on exposures and potential confounders | P17-22, line 182-230 |
|  |  | (b) Indicate number of participants with missing data for each variable of interest | Not relevant |
| Outcome data | 15* | Report numbers of outcome events or summary measures | P17-22, line 182-230 |
| Main results | 16 | (*a*) Give unadjusted estimates and, if applicable, confounder-adjusted estimates and their precision (eg, 95% confidence interval). Make clear which confounders were adjusted for and why they were included | Not relevant |
|  |  | (*b*) Report category boundaries when continuous variables were categorized | Not relevant |
|  |  | (*c*) If relevant, consider translating estimates of relative risk into absolute risk for a meaningful time period | Not relevant |
| Other analyses | 17 | Report other analyses done—eg analyses of subgroups and interactions, and sensitivity analyses | Not relevant |
| Discussion | | |  |
| Key results | 18 | Summarise key results with reference to study objectives | P28 line 300-302 |
| Limitations | 19 | Discuss limitations of the study, taking into account sources of potential bias or imprecision. Discuss both direction and magnitude of any potential bias | P29, 303-307 |
| Interpretation | 20 | Give a cautious overall interpretation of results considering objectives, limitations, multiplicity of analyses, results from similar studies, and other relevant evidence | P28,29, line 301-305 |
| Generalisability | 21 | Discuss the generalisability (external validity) of the study results | P28,29, line 301-307 |
| Other information | | |  |
| Funding | 22 | Give the source of funding and the role of the funders for the present study and, if applicable, for the original study on which the present article is based | P31, line 324-325 |

*Give information separately for exposed and unexposed groups.

**Note:** An Explanation and Elaboration article discusses each checklist item and gives methodological background and published examples of transparent reporting. The STROBE checklist is best used in conjunction with this article (freely available on the Web sites of PLoS Medicine at http://www.plosmedicine.org/, Annals of Internal Medicine at http://www.annals.org/, and Epidemiology at http://www.epidem.com/). Information on the STROBE Initiative is available at www.strobe-statement.org.
